# Bcl-2 protein: a prognostic factor inversely correlated to p53 in non-small-cell lung cancer.

**DOI:** 10.1038/bjc.1995.193

**Published:** 1995-05

**Authors:** G. Fontanini, S. Vignati, D. Bigini, A. Mussi, M. Lucchi, C. A. Angeletti, F. Basolo, G. Bevilacqua

**Affiliations:** Institute of Pathology, University of Pisa, Italy.

## Abstract

**Images:**


British Journal of Cancer (1995) 71, 1003-1007

© 1995 Stockton Press All rights reserved 0007-0920/95 $12.00

### Bcl-2 protein: a prognostic factor inversely correlated to p53 in non-small-cell lung cancer

G Fontanini[1], S Vignati[1], D Bigini[1], A Mussi[2], M Lucchi[2], CA Angeletti[2], F Basolo[1] and G Bevilacqua[1]

[1]*Institute of Pathology and* [2]*Service of Thoracic Surgery, University of Pisa, Pisa, Italy*

**Summary** Non-small-cell lung cancer (NSCLC) prognosis is strictly related to well-established clinicopathological parameters which have unfortunately become insufficient in the prognostic evaluation of this type of cancer. As p53 and *bcl*-2 gene deregulations are frequently involved in several types of epithelial malignancies, we investigated the Bcl-2 and p53 protein expression in 91 and 101 cases of NSCLC respectively. The expression was then compared with established indicators of prognosis and biological behaviour of the tumours. No relationship was observed between Bcl-2 and either clinicopathological or biological parameters such as histology, grading, tumour status, nodal metastasis and proliferative activity evaluated by scoring proliferating cell nuclear antigen expression and Ki-67 immunoreactivity. However, the mean Bcl-2 expression was significantly lower in patients who developed metastasis during follow-up or died of metastatic disease ($P = 0.006$ and $P = 0.01$ respectively). Moreover, survival probability was higher in patients who expressed the Bcl-2 protein ($P = 0.0002$). In contrast with this, p53 protein accumulation was observed in tumours with metastatic nodal involvement ($P = 0.02$) or in patients who developed metastasis during follow-up ($P = 0.01$), although no correlation was found between p53 expression and overall survival. An inverse relationship was also found between Bcl-2 and the anti-oncogene protein product p53 ($P = 0.01$). Thus, a high proportion of NSCLCs express p53 and Bcl-2 proteins and their expression may have prognostic importance.

**Keywords:** oncogenes; NSCLC; prognosis; p53; Bcl-2

Lung cancer has now become the leading cause of cancer deaths in both men and women in the USA (Minna *et al.*, 1989). In particular, non-small-cell lung cancer (NSCLC) represents a heterogeneous subgroup in terms of both behaviour and therapeutic response.

Several studies have clearly demonstrated that multiple genetic events are associated with the development of lung cancer, including a range of chromosomal abnormalities, mutations activating the dominant cellular proto-oncogenes and genetic events inactivating tumour suppressor genes (Minna, 1993).

p53 alterations and aberrant nuclear accumulation of this protein have been recently studied with particular interest. Many experimental data indicate that the p53-suppressor gene is the most commonly altered tumour-suppressor gene (Hollstein *et al.*, 1991). This gene codes for a nuclear phosphoprotein, normally undetectable in human cells, which is able to regulate cell growth and division (Levine *et al.*, 1991; Lane, 1992). p53 protein may be detected by immunocytochemistry in cancer cells as a consequence either of mutational events in p53 gene or of stabilisation by other factors such as some viral proteins. p53 alterations appear early during NSCLC progression; these alterations are maintained during invasion and metastatic spread of cancer cells (Quinlan *et al.*, 1992; Fontanini *et al.*, 1994), inducing a proliferative advantage and conferring a particularly aggressive phenotype.

The *bcl*-2 gene was originally discovered owing to its involvement in the t(14;18) chromosomal translocation occurring in the majority of non-Hodgkin's B-cell lymphomas (Tsujimoto and Croce, 1986; Aisemberg *et al.*, 1988). This translocation places the *bcl*-2 gene at chromosomal location 18q21 in juxtaposition with the immunoglobulin heavy-chain locus at 14q32, resulting in transcriptional deregulation of the *bcl*-2 gene (Cleary *et al.*, 1984; Tsujimoto and Croce, 1986; Tsujimoto *et al.*, 1987) and abnormally high levels of the Bcl-2 protein. Furthermore, overexpression of the Bcl-2 protein has been observed in different types of solid tumours, including prostate (Colombel *et al.*, 1993), lung (Pezzella *et al.*, 1993*a*), thyroid (Pilotti *et al.*, 1994) and breast (Silvestrini *et al.*, 1994). In contrast with lymphomas, little or no evidence of gross alterations in the *bcl*-2 gene structure was obtained for these other types of cancer, suggesting that alternative mechanisms of deregulation of Bcl-2 expression may exist in human cancer (Leek *et al.*, 1994).

The high incidence of p53 alterations and the aberrant expression of Bcl-2 in many human cancers together with their putative prognostic significance in lung (Pezzella *et al.*, 1993*a*) and breast cancer (Silvestrini *et al.*, 1994) induced us to study the p53 and Bcl-2 expression in a series of NSCLCs, with particular regard to their relationship, according to metastatic assessment and overall survival.

### Materials and methods

### *Patients and tissue samples*

The study involved 101 patients with primary resectable non-small-cell lung cancer. The patients (91 men and ten women, mean age 63 ± 6.4 years) presented no clinical or radiological evidence of distant metastases at diagnosis and underwent lobectomy or pneumonectomy between March 1991 and December 1992 at Santa Chiara Hospital of Pisa University. The median follow-up was 25 months (range 2-41). The histopathological features of the surgical specimens were classified and staged according to the World Health Organization (1982) criteria and the TNM staging system (Mountain, 1987). Immediately after surgery a part of the tumour sample was processed by conventional histological procedures for the determination of the Bcl-2 and proliferation cell nuclear antigen (PCNA) expression. The rest of the tumour material was frozen in liquid nitrogen and stored at − 80°C for p53 and Ki-67 determination.

### *Immunohistochemistry*

A total of 101 and 91 samples were examined for p53 and Bcl-2 expression respectively. In 90 cases both p53 and Bcl-2 immunoreactions were performed.

Correspondence: G Fontanini, Institute of Pathology, University of Pisa, 57 Via Roma, 56126 Pisa, Italy
Received 21 September 1994; revised 3 January 1995; accepted 4 January 1995

*Bcl-2 immunostaining* The 5 µm tumour sections were immunostained using the alkaline phosphatase-anti-alkaline phosphatase (APAAP) method (Cordell *et al.*, 1984) with the anti-Bcl-2 monoclonal antibody (clone 124) (Pezzella *et al.*, 1992) raised to a synthetic peptide. Briefly, paraffin sections were dewaxed in xylene and dehydrated through graded alcohols. The monoclonal antibody Bcl-2 124 was applied overnight at 1:20 dilution. A rabbit anti-mouse secondary antibody prediluted in 0.05 M Tris buffer containing normal swine serum was applied for half an hour. The alkaline phosphatase-mouse anti-alkaline phosphatase immune complex was applied for half an hour first and then for 10 min; during the interval the anti-mouse serum was used. The reaction was developed with alkaline phosphatase substrate containing naphthol AS-MX fast red Tr and levamisole (APAAP Kits, Dako). As a positive control for Bcl-2, we used a paraffin-embedded section from a normal peribronchial lymph node removed during post-surgical sampling of a lung tumour. At the same time positive staining of small lymphocytes provided an internal control for Bcl-2 staining. Staining without anti-Bcl-2 monoclonal antibody was performed as a negative control procedure. The Bcl-2 immunoreactivity was assessed by scoring a minimum of five high-power fields (HPFs) (40 × objective lens).

### *p53 immunostaining*

For p53 detection the anti-p53 monoclonal antibody PAb 1801 (Oncogene Science, Manhasset, NY, USA) was used overnight at 1:200 dilution on 5 µm frozen sections, as previously reported (Fontanini *et al.*, 1993*a*). This antibody reacts specifically with human wild-type and mutant p53 recognising an N-terminal epitope of the protein. The avidin-biotin-peroxidase method was used, developing the immunoreaction with diaminobenzidine. Simultaneous staining of a known $p53^+$ case was employed as a positive control for p53 expression. Incubation of parallel slides omitting the first antibody was performed as the negative control. As for Bcl-2, the number of p53-immunoreactive cells was counted by scoring a minimum of five HPFs (40 × objective lens).

### *PCNA and Ki-67 score*

Proliferative activity in each sample was evaluated using PC10 and Ki-67 MAbs on paraffin-embedded and frozen sections respectively, as previously reported (Fontanini *et al.*, 1993*b*). Absolute counts of PC10 and Ki-67 immunoreactivity were made by scoring a minimum of five HPFs (40 × objective lens); 1% PC10- and Ki-67-positive tumour cells out of the total number of tumour cells counted provided the PCNA and Ki-67 index for each tumour.

### *Statistical analysis*

All statistical analyses were carried out by the STATISTICA (Stat-Soft) software system. The differences between p53 and Bcl-2 expression and clinicopathological parameters were assessed by the unpaired *t*-test. The relationship between the p53 and Bcl-2 expression was evaluated by a chi-square test and by a linear regression coefficient test. Survival analysis was calculated by the Kaplan-Meier method.

### Results

### *Bcl-2 protein immunostaining*

Bcl-2 immunoreactivity was localised in the cytoplasm of neoplastic cells (Figure 1a); no nuclear Bcl-2 positivity was found in this series of cancers. We evaluated as positive tumours with only 1% of stained cells, provided this positivity was very defined and localised in the cytoplasm of neoplastic cells. Heterogeneous staining was sometimes detected in the basal layer cells of the normal bronchial epithelium adjacent to tumour areas. The frequency of cells

**Figure 1** (**a**) Bcl-2 expression in the cytoplasm of tumour cells (anti-Bcl-2 MAb, APAAP method, 25 ×). (**b**) p53 nuclear immunoreactivity detected in neoplastic cells using PAb 1801 (ABC method, 25 ×).

expressing the Bcl-2 protein varied widely from one tumour to the other. Bcl-2 protein immunopositivity was detected in 61 out of the 91 (67%) tumour samples examined, ranging from 1.5% to 90% positive cells (mean 27.1 ± 25.1; median 15).

*Bcl-2 expression and clinicopathological parameters* As reported in Table I, no statistical differences in Bcl-2 expression were found between: (1) tumour histotype, (2) tumour grade, (3) tumour status and (4) nodal metastasis.

*Bcl-2 expression and proliferative activity* Highly proliferating tumours (cut-off 30% for PCNA and 13% for Ki-67) express a percentage of positive cells similar to that of tumours with low proliferative activity (Table II). These results suggest that the Bcl-2 protein expression is independent of the proliferative status of the tumours.

*Bcl-2 expression in relation to distant metastasis and survival* In 89 out of 91 patients the data regarding development of metastases and overall survival were available (two patients died during the post-operative period). In order to determine whether the alterations in the Bcl-2 protein may be a prognostic indicator in NSCLC, we analysed Bcl-2 expression both in tumours from patients with and without metastases and in patients still alive or dead from neoplastic disease. Bcl-2 expression was significantly higher ($P < 0.006$) in tumours from metastasis-free patients than in tumours from patients with distant metastases. We also found that tumours from living patients presented a higher number of Bcl-2-immunoreactive cells than tumours from dead patients ($P < 0.01$) (Table III). The same results obtained from survival analysis are reported in Figure 2a. NSCLC patients with Bcl-2-positive tumours had a higher probability of survival than patients with Bcl-2-negative cancers (Kaplan-Meier analysis, $P = 0.0002$). We obtained the same results

**Table I** Clinicopathological data and immunocytochemical reactivity for Bcl-2 protein in 91 cases of NSCLC

| *Variables* | *No. of cases* | *Bcl-2 immunoreactivity* *Mean (± s.d.)* | P[a] |
|---|---|---|---|
| Sex | | | |
| Male | 81 | 17.6 ± 23.7 | NS |
| Female | 10 | 22.9 ± 28.8 | |
| Histology | | | |
| Squamous | 55 | 19.9 ± 23.2 | NS |
| Non-squamous | 36 | 15.4 ± 26.6 | |
| Grading | | | |
| G1 | 16 | 17.7 ± 24.4 | |
| G2 | 38 | 18.1 ± 23.5 | NS |
| G3 | 37 | 17.1 ± 251 | |
| T-status | | | |
| T1 | 16 | 22.8 ± 23.8 | |
| T2 | 64 | 17.4 ± 24.2 | NS |
| T3 | 11 | 15.7 ± 25.7 | |
| N-status | | | |
| N0 | 64 | 17.5 ± 21.8 | NS |
| N1-2 | 27 | 19.6 ± 29.4 | |

[a]Unpaired *t*-test.

**Table II** Bcl-2 protein expression in NSCLC according to PCNA and Ki-67 immunoreactivity

| *Proliferative activity* | *No. of cases* | *Bcl-2 immunoreactivity* *Mean (± s.d)* | P[a] |
|---|---|---|---|
| PCNA | | | |
| ≤ 30[a] | 33 | 15.3 ± 25.9 | 0.6 |
| > 30 | 26 | 18.1 ± 26.5 | |
| Ki-67 | | | |
| < 13[a] | 35 | 17.6 ± 27.8 | 0.6 |
| > 13 | 24 | 15.6 ± 24.7 | |

[a]Unpaired *t*-test.

**Table III** Relationship between Bcl-2 protein expression, survival and metastasis in 89 patients with NSCLC

| | *No. of cases* | *Bcl-2 expression* *Mean (± s.d)* | P[a] |
|---|---|---|---|
| Alive | 51 | 22.8 ± 24.2 | 0.01 |
| Dead | 38 | 10.8 ± 22.3 | |
| No metastasis | 48 | 24.2 ± 24.4 | 0.006 |
| Metastasis | 41 | 10.2 ± 21.5 | |

[a]Unpaired *t*-test.

when considering Bcl-2 expression as a dichotomous variable (negative *vs* positive tumours) (Table IV). Taken together, these results suggest that Bcl-2 expression may be considered as a favourable prognostic marker in this type of cancer.

### *p53 protein immunostaining*

p53 immunostaining was performed in 101 cases. Sixty-nine cases were found to be positive and 32 negative (mean 39.9 ± 28.5; range 1-85). Immunoreactivity was confined to the nuclei of neoplastic cells (Figure 1b) with the following staining pattern: (a) tumours with only a few scattered positive cells (< 1%) or none at all were considered negative; tumours with more than 1% positive cells with either (b) heterogeneous or (c) homogeneous distribution were evaluated as positive.

*p53 expression and clinicopathological parameters* The mean p53 immunoreactivity according to clinicopathological parameters is summarised in Table V. As is shown, no correlation was found between p53 expression and clinicopathological parameters such as T status, histiotype and tumour grade. By contrast, mean p53 immunoreactivity in tumours from patients with hilar and/or mediastinal nodal involvement was significantly higher than in patients without nodal metastasis ($P = 0.02$).

**Figure 2** Survival of patients with non-small-cell lung cancer according to status of Bcl-2. (**a**) and p53 (**b**) proteins (Kaplan-Meier method).

**Table IV** Survival and metastasis in 89 patients with NSCLC according to Bcl-2 expression

| | *Bcl-2 expression* *Positive cases/ total* | *Negative cases/ total* | P[a] |
|---|---|---|---|
| Alive | 42 | 9 | 0.0005 |
| Dead | 17 | 21 | |
| No metastasis | 40 | 8 | 0.0006 |
| Metastasis | 19 | 22 | |

[a]Contingency tables.

**Table V** p53 immunoreactivity in 101 cases of NSCLC according to clinicopathological parameters

| *Variables* | *No. of cases* | *p53 immunoreactivity* *Mean (± s.d.)* | P[a] |
|---|---|---|---|
| Sex | | | |
| Male | 91 | 27.9 ± 27.6 | NS |
| Female | 10 | 21.1 ± 33.3 | |
| Histology | | | |
| Squamous | 64 | 24.7 ± 27.4 | NS |
| Non-squamous | 37 | 31.6 ± 29.2 | |
| Grading | | | |
| G1 | 17 | 24.7 ± 28.9 | |
| G2 | 43 | 26.1 ± 27.7 | NS |
| G3 | 41 | 31.1 ± 28.7 | |
| T-status | | | |
| T1 | 17 | 25.4 ± 30.6 | |
| T2 | 73 | 27.7 ± 27.4 | NS |
| T3 | 11 | 22.04 ± 31.8 | |
| N-status | | | |
| N0 | 62 | 22.9 ± 26.6 | 0.02 |
| N1-2 | 39 | 36.6 ± 29.4 | |

[a]Unpaired *t*-test.

Table VI p53 expression in NSCLC according to the development of metastasis and survival

| | No. of cases | p53 immunoreactivity Mean ( ± s.d) | P* |
|---|---|---|---|
| Metastasis | 44 | 35.4 ± 28.3 | 0.01 |
| No metastasis | 55 | 21.7 ± 26.7 | |

*Unpaired t-test.

Table VII Bcl-2 and p53 expression in 59 cases of non-small cell lung cancer

| p53 expression | Bcl-2 expression No. of positive cases/Total | % | No. of negative cases/Total | % | P* |
|---|---|---|---|---|---|
| p53 positive | 36 | 60 | 26 | 86.7 | 0.01 |
| p53 negative | 24 | 40 | 4 | 13.3 | |

*Contingency tables.

Figure 3 Relation between Bcl-2 and p53 expression in 90 cases of non-small-cell lung cancer (linear regression coefficient test).

*p53 expression in relation to distant metastases and survival*

In 99 out of 101 cases we obtained data concerning either the development of metastases during follow-up or overall survival (two patients died during the post-operative period). Forty-four out of 99 (44.4%) patients who had developed distant metastasis showed a higher mean p53 positivity (35.4%) than metastasis-free patients (21.7%) ($P = 0.01$; Table VI). On the other hand, overall survival was not affected by p53 overexpression (Figure 2b; Kaplan–Meier method).

*Bcl-2 and p53 protein expression*

Staining for both Bcl-2 and p53 was available in 90 cases. The results are summarised in Table VII. Of 60 Bcl-2-positive tumours, 60% showed p53 overexpression, whereas of 30 Bcl-2-negative tumours 87% showed p53 immunoreactivity ($P = 0.01$). Regression analysis is reported in Figure 3; a clear inverse correlation was found between the p53 and Bcl-2 protein expression ($P = 0.01$).

### Discussion

Bcl-2 and p53 proteins are both related to programmed cell death or apoptosis and thus their relationship is of interest. In this series of NSCLCs the results showed that Bcl-2 and p53: (a) are detectable by the immunohistochemical technique in about 60% and 70% of cases respectively; (b) are inversely associated; and (c) provide information regarding metastasis onset and overall survival probability.

Bcl-2 represents the product of the proto-oncogene involved in the 14;18 translocation; its distribution in reactive lymph nodes and lymphomas has already been described (Villeundas *et al.*, 1992; Pezzella *et al.*, 1993*b*; Piris *et al.*, 1994). Bcl-2 expression has been observed not only in the B-lymphoma but also in different types of solid tumours such as breast (Leek *et al.*, 1994; Silvestrini *et al.*, 1994), prostate (Colombel *et al.*, 1993) thyroid (Pilotti *et al.*, 1994) and lung cancer (Pezzella *et al.*, 1993*a*).

In particular, in NSCLCs, which are believed to originate from the respiratory epithelium, Bcl-2 overexpression was related with better overall survival (Pezzella *et al.*, 1993*a*). Our results obtained from a group of 91 NSCLC patients with median follow-up of 24 months agree with those reported by Pezzella *et al.* However, both studies found that Bcl-2 overexpression seems to be able to induce a less aggressive tumour phenotype. The reason for this remains to be clarified. Interestingly, in other types of cancers such as breast (Leek *et al.*, 1994; Silvestrini *et al.*, 1994), thyroid (Pilotti *et al.*, 1994) and prostate (Colombel *et al.*, 1994) a strong association has been found between Bcl-2 expression and tumour differentiation, suggesting that this gene may somehow act to switch off proliferation during tumour progression. However, this hypothesis cannot be confirmed in lung cancer since we failed to find any differences in the Bcl-2 expression of tumours of different grade. It is known that Bcl-2 promotes cell survival even when the cell proliferation rate is not elevated. This could provide a growth advantage eventually leading to neoplastic transformation (Vaux *et al.*, 1988). McDonnel *et al.* (1989) suggested that for cellular clones in which a low proliferative rate is offset by Bcl-2 expression the acquisition rate of complementary defects is slower than in clones with a higher proliferative rate.

The association of a growth advantage owing to cellular survival with low proliferative rate and slower acquisition of further genetic defects could explain the slow evolution of follicular lymphoma in which Bcl-2 expression is a frequent primary aberration (Vaux *et al.*, 1988; McDonnel *et al.*, 1989). Different authors have suggested recently that alterations in Bcl-2 could be present as a frequent aberration not only in follicular lymphoma (Korsmeyer, 1992), but also in other types of cancer such as breast (Silvestrini *et al.*, 1994; Leek *et al.*, 1994) and lung (Pezzella *et al.*, 1993*a*) carcinomas. This relation could be responsible for the increasing likelihood of mutational aberrations in other oncogenes, such as those interfering with growth and proliferation of tumour cells.

Our observations of a subgroup of patients with slowly progressing Bcl-2-positive tumours suggest that in these tumours Bcl-2 expression is likely to occur as an initial alteration leading to a less aggressive behaviour of tumours. This is in agreement with the inverse relationship between Bcl-2 and p53 which we found in our series of cancers, supporting the hypothesis that either one or the other is sufficient to modify the apoptotic pathway in NSCLC.

In our study Bcl-2 expression is not associated with cell proliferation evaluated as a percentage of PC10- or Ki-67-immunoreactive cells. In addition, we found that the bronchial epithelium expresses Bcl-2 in a proportion of basal cells, and that there is a total lack of Bcl-2-positive cells in the upper differentiated layers of the epithelium although hyperplasia is sometimes present in these areas. These findings have been confirmed by Lu *et al.* (1993), who have recently reported the lack of Bcl-2 expression in non-proliferating syncytiotrophoblast and in psoriasis despite evident mitotic activity. This suggests that Bcl-2 is mainly associated with activated and undifferentiated cells undergoing terminal differentiation which need protection from apoptosis.

p53 is now well characterised as a tumour-suppressor gene, with loss of normal p53 function recorded as the most common genetic event associated with human malignancies (Hollstein, 1991). p53 alterations with consequent aberrant nuclear accumulation have been correlated with progression and poor prognosis in some solid tumours such as breast (Thor et al., 1992; Allred et al., 1993; Silvestrini et al., 1993), gastric (Martin et al., 1992), bladder (Sarkis et al., 1993) and lung (Quinlan et al., 1992) carcinomas. In a series of 103 NSCLCs (Fontanini et al., 1993a) we found that p53 protein overexpression correlated with metastatic involvement of hilar and/ or mediastinal lymph nodes, supporting other findings by Quinlan et al. about the negative prognostic role of p53 alterations in NSCLC. In this group of patients it was confirmed that p53 accumulation may predict the metastatic behaviour of NSCLC, and that it is overexpressed not only in cancer with nodal metastatic involvement at diagnosis but also in tumours which develop distant metastases during follow-up.

Our present results indicate on the one hand an inverse relationship between Bcl-2 and p53 expression and, on the other hand, an inverse prognostic significance of these variables in NSCLC behaviour. The loss of Bcl-2 expression is in fact associated with shorter overall survival, metastatic development during follow-up and other poor prognostic markers such as p53 positivity. For this reason, the role of Bcl-2 in lung cancer progression may differ from that seen in lymphomas in which the translation 14;18 occurs.

Further efforts are needed to assess the prognostic significance of Bcl-2 and its relation with other gene products involved in the regulation of apoptosis and proliferation.

**Acknowledgement**
This work was supported by AIRC (Italian Association for Cancer Research) and by CNR PF ACRO project, Contract No. 94.01081.PF39. Dr D Bigini is an AIRC fellow.

### References

AISEMBERG AC, WILKES BM AND JACOBSON JO. (1988). The bcl2 gene is rearranged in many diffuse B-cell lymphomas. *Blood*, **71**, 969-972.

ALLRED DC, CLARK GM, ELLEDGE R, *et al.* (1993). Association of p53 protein expression with tumor cell proliferation rate and clinical outcome in node-negative breast cancer. *J. Natl Cancer Inst.*, **85**, 200-206.

CLEARY ML, SMITH SD AND SKLAR J. (1984). Cloning and structural analysis of cDNAs for bcl2 and a hybrid bcl2/ immunoglobulin transcript resulting from the t(14;18) transcription. *Science*, **226**, 1097-1099.

COLOMBEL M, SYMMANS F, GIL S, O'TOOLE KM, *et al.* (1993). Detection of the apoptosis-suppressing oncoprotein bcl-2 in hormone-refractory human prostate cancers. *Am. J. Pathol.*, **143**, 390-400.

CORDELL JL, FALINI B, ERBER WN, *et al.* (1984). Immunoenzymatic labelling of monoclonal antibodies using immune complexes of alkaline phosphatase and monoclonal anti-alkaline phosphatase (APAAP complex). *J. Histochem. Cytochem.*, **32**, 219-222.

FONTANINI G, BIGINI D, VIGNATI S, *et al.* (1993*a*). p53 expression in non small cell lung cancer: clinical and biological correlations. *Anticancer Res.*, **13**, 737-742.

FONTANINI G, PINGITORE R, BIGINI D, VIGNATI S, PEPE S, RUGGIERO A AND MACCHIARINI P. (1993*b*). Growth fraction in non small cell lung cancer estimated by proliferating cell nuclear antigen and comparison with Ki-67 labelling and DNA flow cytometry data. *Am. J. Pathol.*, **141**, 1285-1290.

FONTANINI G, VIGNATI S, BIGINI D, MERLO GR, RIBECCHINI A, ANGELETTI CA, BASOLO F, PINGITORE R AND BEVILACQUA G. (1994). Human non small cell lung cancer: p53 accumulation is an early event and persists during metastatic progression. *J. Pathol.*, **174**, 23-31.

HOLLSTEIN M, SIDRANSKY D, VOGELSTEIN B AND HARRIS CC. (1991). P53 mutation in human cancers. *Science*, **253**, 49-53.

KORSMEYER SJ. (1992). BCL2 initiates a new category of oncogenes: regulator of cell death. *Blood*, **80**, 876-879.

LANE DP (1992). P53, guardian of the genome. *Nature*, **358**, 15-16.

LEEK RD, KAKLAMANIS L, PEZZELLA F, GATTER KC AND HARRIS AL. (1994). Bcl-2 in normal human breast and carcinoma, association with oestrogen receptor-positive, epidermal growth factor receptor-negative tumors and *in situ* cancer. *Br. J. Cancer*, **69**, 135-139.

LEVINE AJ, MOMAND J AND FINLEY CA. (1991). The p53 tumour suppressor gene. *Nature*, **351**, 453-456.

LU QL, POULSOM R, WONG L AND HANBY AM. (1993). Bcl-2 expression in adult and embryonic non-haematopoietic tissue. *J. Pathol.*, **169**, 431-437.

MARTIN HM, FILIPE MI, MORRIS RW, LANE DP AND SILVESTRE F. (1992). P53 expression and prognosis in gastric carcinoma. *Int. J. Cancer*, **50**, 859-862.

McDONNEL TI, DEANE N, PLATT FM, *et al.* (1989). BCL2 immunoglobulin transgenic mice demonstrate extended B cell survival and follicular lymphoproliferation. *Cell*, **57**, 79-88.

MINNA JD, PASS H, GALSTEIN E AND IHDE D. (1989). Cancer of the lung. In *Cancer, Principles and Practice of Oncology*, Devita VT, Hellman S, Rosemberg SA. (eds) pp. 591-705. Philadelphia: JB Lippincott.

MINNA JD (1993). The molecular biology of lung cancer pathogenesis. *Chest*, **103**, 445S-56S.

MOUNTAIN CF. (1987). The new international staging system for lung cancer. *Surg. Clin. N. Am.*, **67**, 925-935.

PEZZELLA F, JONES M, RALFKIER E, ERSBØL J, GATTER KC AND MASON DJ. (1992). Evaluation of bcl2 protein expression and 14;18 translocation as prognostic markers in follicular lymphoma. *Br. J. Cancer*, **65**: 87-89.

PEZZELLA F, TURLEY H, KUZU I, *et al.* (1993*a*). Bcl-2 protein in non small-cell lung carcinoma. *N. Engl. J. Med.*, **329**, 690-694.

PEZZELLA F, MORRISON H, JONES M, GATTER KC, LANE C, HARRIS AL AND MASON DY. (1993*b*). Immunohistochemical detection of p53 and bcl2 proteins in non-Hodgkin's lymphoma. *Histopathology*, **22**, 39-44.

PILOTTI S, COLLINI P, RILKE F, CATTORETTI G, DEL BO R. AND PIEROTTI MA. (1994). Bcl-2 protein expression in carcinomas originating from the follicular epithelium of the thyroid gland. *J. Pathol.*, **172**, 337-342.

PIRIS MA, PEZZELLA F, MARTINEZ-MONTERO JC, *et al.* (1994). p53 and bcl2 expression in high-grade B-cell lymphomas: correlation with survival time. *Br. J. Cancer*, **69**, 337-341.

QUINLAN DC, DAVIDSON AS, SUMMERS CL, WARDEN HE AND DOSHI HM. (1992). Accumulation of p53 correlates with a poor prognosis in human lung cancer. *Cancer Res.*, **52**, 4828-4831.

SARKIS AS, DALBAGNI G, CORDON-CARDO C, *et al.* (1993). Nuclear overexpression of p53 protein in transitional cell bladder carcinoma: a marker for disease progression. *J. Natl Cancer Inst.*, **85**: 53-59.

SILVESTRINI R, BENINI E, DAIDONE MG, et al. (1993). P53 as an independent prognostic marker in lymph node-negative breast cancer patients. *J Natl Cancer Inst.*, **85**, 965-970.

SILVESTRINI R, VENERONI S, DAIDONE MG, *et al.* (1994). The bcl-2 protein: a prognostic indicator strongly related to p53 protein in lymph node-negative breast cancer patients. *J. Natl Cancer Inst.*, **86**, 499-504.

THOR AD, MOORE DH, EDGERTON SM, *et al.* (1992). Accumulation of p53 tumor suppressor gene protein: an independent marker of prognosis in breast cancers. *J. Natl Cancer Inst.*, **84**, 845-855.

TSUJIMOTO J AND CROCE CM. (1986). Analysis of the structure, transcripts and protein products of BCL2, the gene involved in human follicular lymphoma. *Proc. Natl Acad. Sci. (USA)*, **83**, 5214-5218.

TSUJIMOTO J, IKEGAKI N AND CROCE CM. (1987). Characterization of the protein product of BCL2, the gene involved in human follicular lymphoma. *Oncogene*, **2**, 3-7.

VAUX DL, CORY S AND ADAMS JM. (1988). BCL2 gene promotes haemopoietic cell survival and cooperates with C-myc immortalized pre-B cells. *Nature*, **335**, 440-442.

VILLEUNDAS R, PIRIS MA, ORRADRE JL, MOLLEJO M, ALGARA P, SANCHEZ L, MARTINEZ JC AND MARTINEZ P. (1992). p53 protein expression in lymphomas and reactive lymphoid tissue. *J. Pathol.*, **166**, 235-241.

WORLD HEALTH ORGANIZATION. (1982). The world health organization histological typing of lung cancer. *Am. J. Clin Pathol.*, **77**, 123-136.


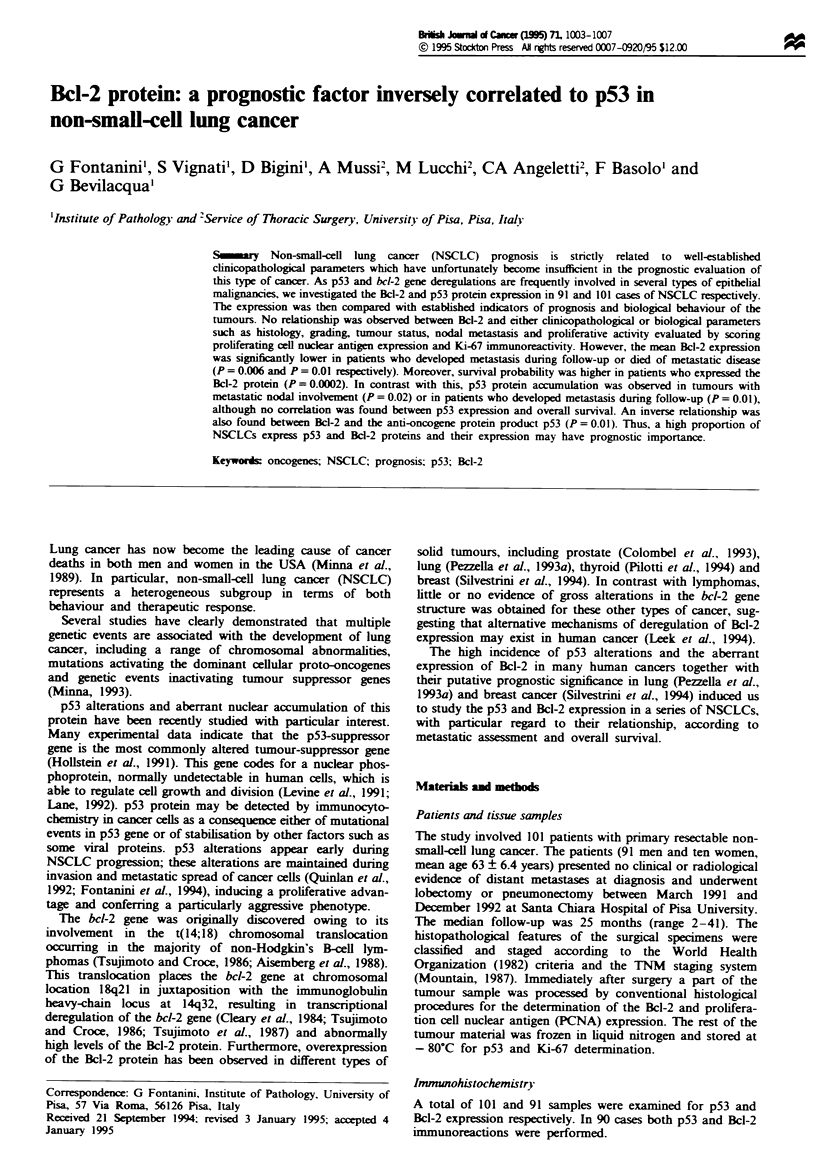

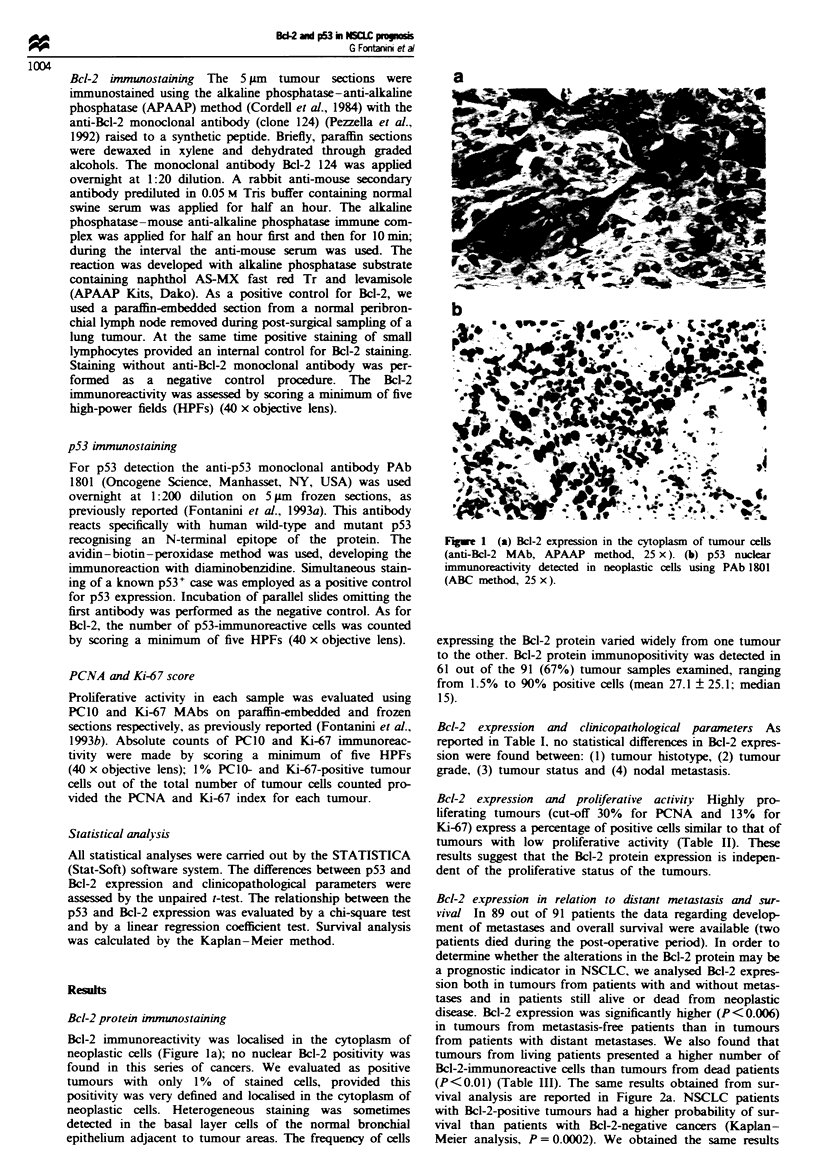

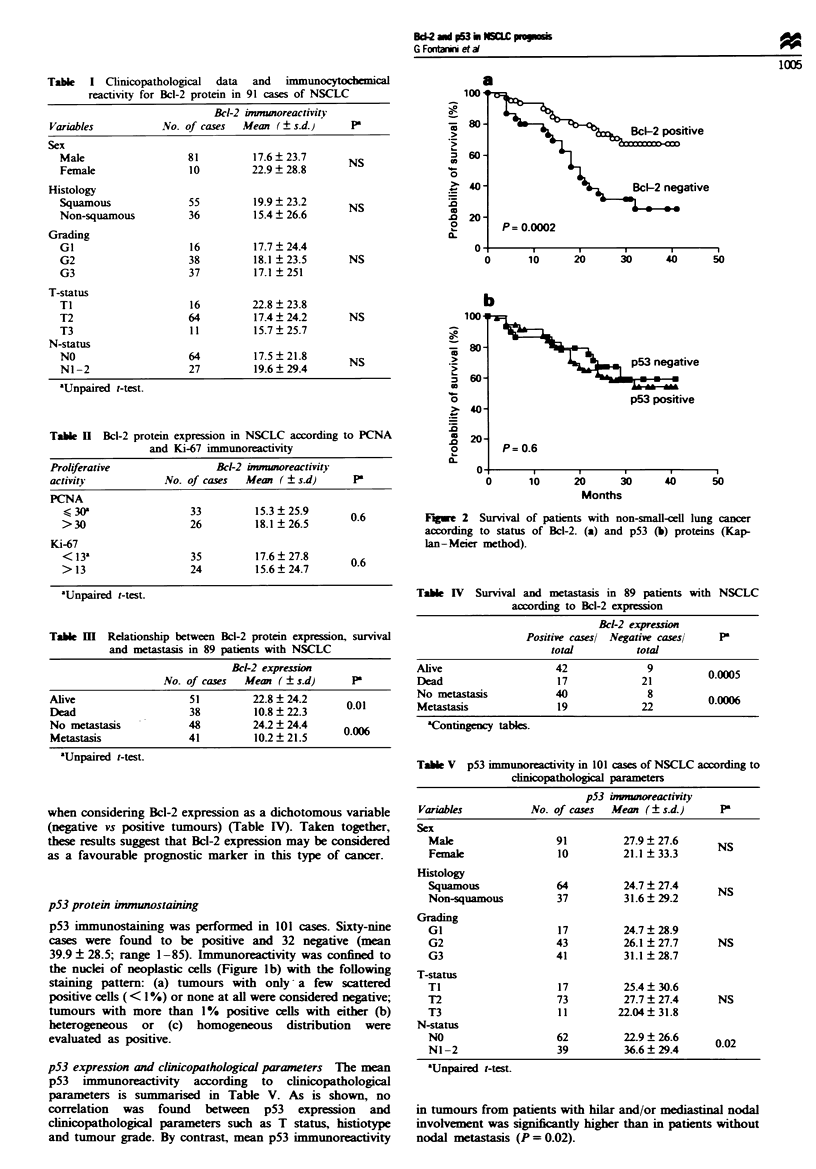

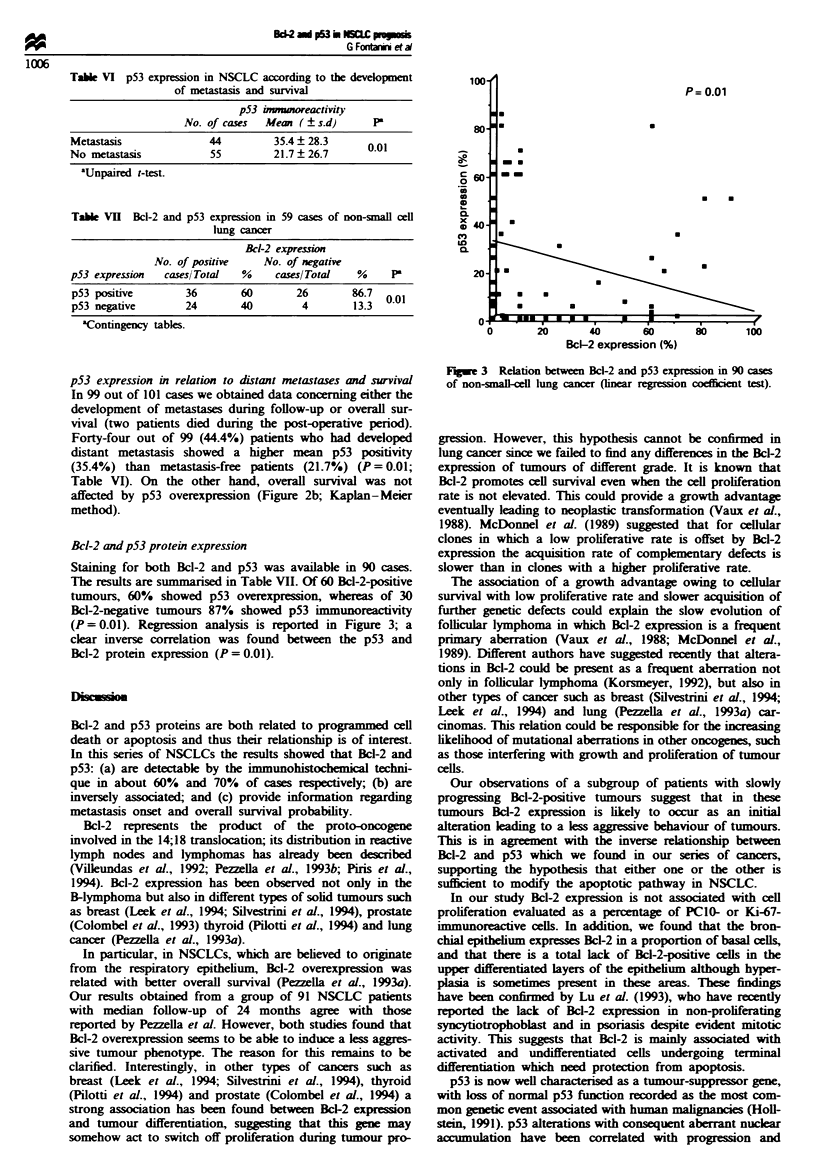

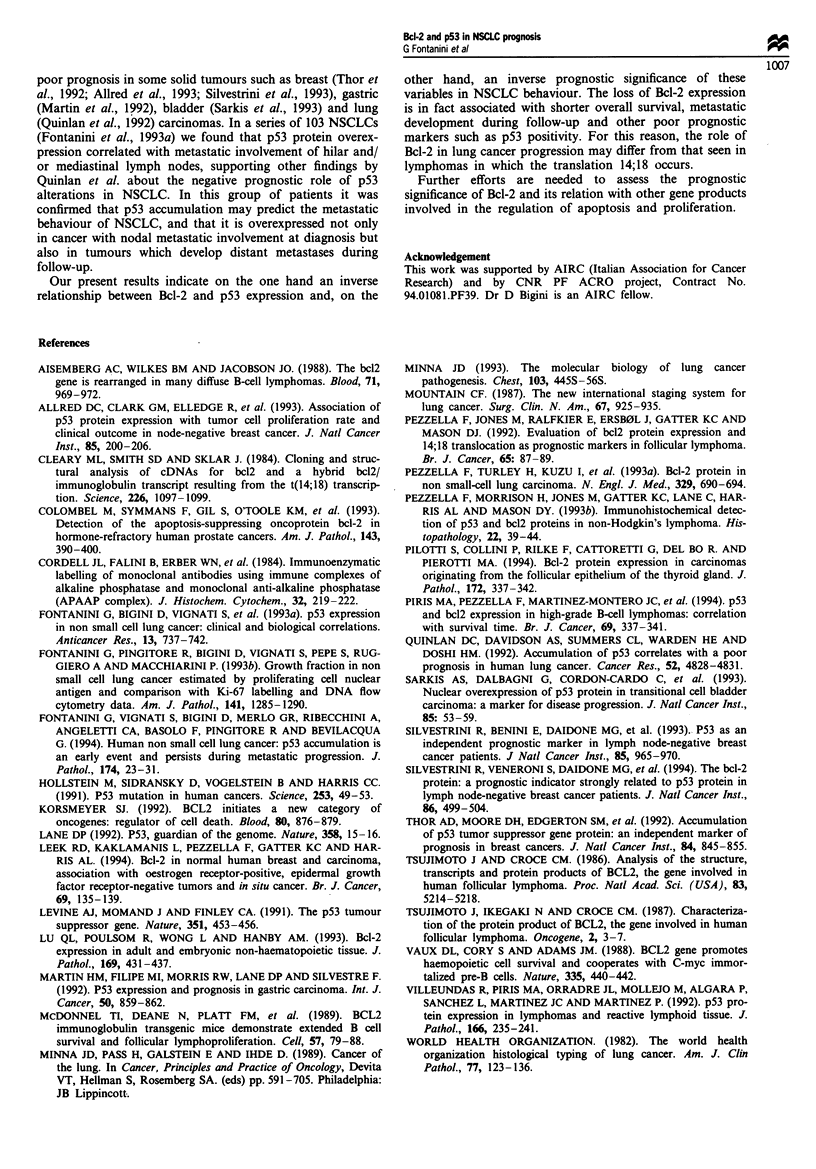


## References

[OCR_00689] Aisenberg A. C., Wilkes B. M., Jacobson J. O. (1988). The bcl-2 gene is rearranged in many diffuse B-cell lymphomas.. Blood.

[OCR_00694] Allred D. C., Clark G. M., Elledge R., Fuqua S. A., Brown R. W., Chamness G. C., Osborne C. K., McGuire W. L. (1993). Association of p53 protein expression with tumor cell proliferation rate and clinical outcome in node-negative breast cancer.. J Natl Cancer Inst.

[OCR_00706] Colombel M., Symmans F., Gil S., O'Toole K. M., Chopin D., Benson M., Olsson C. A., Korsmeyer S., Buttyan R. (1993). Detection of the apoptosis-suppressing oncoprotein bc1-2 in hormone-refractory human prostate cancers.. Am J Pathol.

[OCR_00712] Cordell J. L., Falini B., Erber W. N., Ghosh A. K., Abdulaziz Z., MacDonald S., Pulford K. A., Stein H., Mason D. Y. (1984). Immunoenzymatic labeling of monoclonal antibodies using immune complexes of alkaline phosphatase and monoclonal anti-alkaline phosphatase (APAAP complexes).. J Histochem Cytochem.

[OCR_00718] Fontanini G., Bigini D., Vignati S., Macchiarini P., Pepe S., Angeletti C. A., Pingitore R., Squartini F. (1993). p53 expression in non small cell lung cancer: clinical and biological correlations.. Anticancer Res.

[OCR_00721] Fontanini G., Pingitore R., Bigini D., Vignati S., Pepe S., Ruggiero A., Macchiarini P. (1992). Growth fraction in non-small cell lung cancer estimated by proliferating cell nuclear antigen and comparison with Ki-67 labeling and DNA flow cytometry data.. Am J Pathol.

[OCR_00728] Fontanini G., Vignati S., Bigini D., Merlo G. R., Ribecchini A., Angeletti C. A., Basolo F., Pingitore R., Bevilacqua G. (1994). Human non-small cell lung cancer: p53 protein accumulation is an early event and persists during metastatic progression.. J Pathol.

[OCR_00735] Hollstein M., Sidransky D., Vogelstein B., Harris C. C. (1991). p53 mutations in human cancers.. Science.

[OCR_00739] Korsmeyer S. J. (1992). Bcl-2 initiates a new category of oncogenes: regulators of cell death.. Blood.

[OCR_00743] Lane D. P. (1992). Cancer. p53, guardian of the genome.. Nature.

[OCR_00744] Leek R. D., Kaklamanis L., Pezzella F., Gatter K. C., Harris A. L. (1994). bcl-2 in normal human breast and carcinoma, association with oestrogen receptor-positive, epidermal growth factor receptor-negative tumours and in situ cancer.. Br J Cancer.

[OCR_00751] Levine A. J., Momand J., Finlay C. A. (1991). The p53 tumour suppressor gene.. Nature.

[OCR_00757] Lu Q. L., Poulsom R., Wong L., Hanby A. M. (1993). Bcl-2 expression in adult and embryonic non-haematopoietic tissues.. J Pathol.

[OCR_00760] Martin H. M., Filipe M. I., Morris R. W., Lane D. P., Silvestre F. (1992). p53 expression and prognosis in gastric carcinoma.. Int J Cancer.

[OCR_00767] McDonnell T. J., Deane N., Platt F. M., Nunez G., Jaeger U., McKearn J. P., Korsmeyer S. J. (1989). bcl-2-immunoglobulin transgenic mice demonstrate extended B cell survival and follicular lymphoproliferation.. Cell.

[OCR_00782] Mountain C. F. (1987). The new International Staging System for Lung Cancer.. Surg Clin North Am.

[OCR_00787] Pezzella F., Jones M., Ralfkiaer E., Ersbøll J., Gatter K. C., Mason D. Y. (1992). Evaluation of bcl-2 protein expression and 14;18 translocation as prognostic markers in follicular lymphoma.. Br J Cancer.

[OCR_00796] Pezzella F., Morrison H., Jones M., Gatter K. C., Lane D., Harris A. L., Mason D. Y. (1993). Immunohistochemical detection of p53 and bcl-2 proteins in non-Hodgkin's lymphoma.. Histopathology.

[OCR_00792] Pezzella F., Turley H., Kuzu I., Tungekar M. F., Dunnill M. S., Pierce C. B., Harris A., Gatter K. C., Mason D. Y. (1993). bcl-2 protein in non-small-cell lung carcinoma.. N Engl J Med.

[OCR_00799] Pilotti S., Collini P., Rilke F., Cattoretti G., Del Bo R., Pierotti M. A. (1994). Bcl-2 protein expression in carcinomas originating from the follicular epithelium of the thyroid gland.. J Pathol.

[OCR_00805] Piris M. A., Pezzella F., Martinez-Montero J. C., Orradre J. L., Villuendas R., Sanchez-Beato M., Cuena R., Cruz M. A., Martinez B., Pezella F [corrected to Pezzella F. ]. (1994). p53 and bcl-2 expression in high-grade B-cell lymphomas: correlation with survival time.. Br J Cancer.

[OCR_00810] Quinlan D. C., Davidson A. G., Summers C. L., Warden H. E., Doshi H. M. (1992). Accumulation of p53 protein correlates with a poor prognosis in human lung cancer.. Cancer Res.

[OCR_00817] Sarkis A. S., Dalbagni G., Cordon-Cardo C., Zhang Z. F., Sheinfeld J., Fair W. R., Herr H. W., Reuter V. E. (1993). Nuclear overexpression of p53 protein in transitional cell bladder carcinoma: a marker for disease progression.. J Natl Cancer Inst.

[OCR_00823] Silvestrini R., Benini E., Daidone M. G., Veneroni S., Boracchi P., Cappelletti V., Di Fronzo G., Veronesi U. (1993). p53 as an independent prognostic marker in lymph node-negative breast cancer patients.. J Natl Cancer Inst.

[OCR_00826] Silvestrini R., Veneroni S., Daidone M. G., Benini E., Boracchi P., Mezzetti M., Di Fronzo G., Rilke F., Veronesi U. (1994). The Bcl-2 protein: a prognostic indicator strongly related to p53 protein in lymph node-negative breast cancer patients.. J Natl Cancer Inst.

[OCR_00834] Thor A. D., Moore DH I. I., Edgerton S. M., Kawasaki E. S., Reihsaus E., Lynch H. T., Marcus J. N., Schwartz L., Chen L. C., Mayall B. H. (1992). Accumulation of p53 tumor suppressor gene protein: an independent marker of prognosis in breast cancers.. J Natl Cancer Inst.

[OCR_00836] Tsujimoto Y., Croce C. M. (1986). Analysis of the structure, transcripts, and protein products of bcl-2, the gene involved in human follicular lymphoma.. Proc Natl Acad Sci U S A.

[OCR_00847] Vaux D. L., Cory S., Adams J. M. (1988). Bcl-2 gene promotes haemopoietic cell survival and cooperates with c-myc to immortalize pre-B cells.. Nature.

[OCR_00852] Villuendas R., Piris M. A., Orradre J. L., Mollejo M., Algara P., Sanchez L., Martinez J. C., Martinez P. (1992). P53 protein expression in lymphomas and reactive lymphoid tissue.. J Pathol.

